# Abuse of Older Men in Seven European Countries: A Multilevel Approach in the Framework of an Ecological Model

**DOI:** 10.1371/journal.pone.0146425

**Published:** 2016-01-19

**Authors:** Maria Gabriella Melchiorre, Mirko Di Rosa, Giovanni Lamura, Francisco Torres-Gonzales, Jutta Lindert, Mindaugas Stankunas, Elisabeth Ioannidi-Kapolou, Henrique Barros, Gloria Macassa, Joaquim J. F. Soares

**Affiliations:** 1 Centre for Socio-Economic Research on Ageing, Italian National Institute of Health and Science on Aging, I.N.R.C.A., Ancona, Italy; 2 Scientific Direction, Italian National Institute of Health and Science on Aging, I.N.R.C.A., Ancona, Italy; 3 Centro de Investigaciones Biomedicas en Red de Salud Mental (CIBERSAM), University of Granada, Granada, Spain; 4 Department of Public Health, University of Emden, Emden, Germany; 5 Women's Studies Research Center, Brandeis University, Waltham, Massachusetts, United States of America; 6 Department of Health Management, Lithuanian University of Health Sciences, Kaunas, Lithuania; 7 Health Service Management Department, Centre for Health Innovation, School of Medicine, Griffith University, Gold Coast, Queensland, Australia; 8 Department of Sociology, National School of Public Health, Athens, Greece; 9 Department of Hygiene and Epidemiology, Medical School, University of Porto, Porto, Portugal; 10 Department of Occupational and Public Health Sciences, University of Gävle, Gävle, Sweden; 11 Department of Public Health Sciences, Karolinska Institute, Stockholm, Sweden; 12 Department of Health Sciences, Section of Public Health Sciences, Mid Sweden University, Sundsvall, Sweden; Texas Tech University Health Science Centers, UNITED STATES

## Abstract

**Background:**

Several studies on elder abuse indicate that a large number of victims are women, but others report that men in later life are also significantly abused, especially when they show symptoms of disability and poor health, and require help for their daily activities as a result. This study focused on the prevalence of different types of abuse experienced by men and on a comparison of male victims and non-victims concerning demographic/socio-economic characteristics, lifestyle/health variables, social support and quality of life. Additionally, the study identified factors associated with different types of abuse experienced by men and characteristics associated with the victims.

**Methods:**

The cross-sectional data concerning abuse in the past 12 months were collected by means of interviews and self-response during January-July 2009, from a sample of 4,467 not demented individuals aged between 60–84 years living in seven European countries (Germany, Greece, Italy, Lithuania, Portugal, Spain and Sweden). We used a multilevel approach, within the framework of an Ecological Model, to explore the phenomenon of abuse against males as the complex result of factors from multiple levels: individual, relational, community and societal.

**Results:**

Multivariate analyses showed that older men educated to higher levels, blue-collar workers and men living in a rented accommodation were more often victims than those educated to lower levels, low-rank white-collar workers and home owners, respectively. In addition, high scores for factors such as somatic and anxiety symptoms seemed linked with an increased probability of being abused. Conversely, factors such as increased age, worries about daily expenses (financial strain) and greater social support seemed linked with a decreased probability of being abused.

**Conclusions:**

Male elder abuse is under-recognized, under-detected and under-reported, mainly due to the vulnerability of older men and to social/cultural norms supporting traditional male characteristics of stoicism and strength. Further specific research on the topic is necessary in the light of the present findings. Such research should focus, in particular, on societal/community aspects, as well as individual and family ones, as allowed by the framework of the Ecological Model, which in turn could represent a useful method also for developing prevention strategies for elder abuse.

## Introduction

Several studies on elder abuse indicate that women are more often victims than men [1–8]. For instance, the United Kingdom (UK) study on mistreatment [1] reported that 3.8% of women and 1.1% of men were victims. When neglect was excluded, the prevalence of abuse continued to be significantly higher for women (2.3%) than men (0.6%). In Ireland, women (2.4%) were more likely than men (1.9%) to report experiences of maltreatment in the previous 12 months, especially concerning interpersonal violence [5]. In particular, women seemed to experience most of the more severe cases of emotional abuse [6]. Also, in care homes, women seemed to be more often abused than men [3].

However, some of the above mentioned studies indicate that men were more financially abused [1] and exposed to aggravated assaults [4] than women. Furthermore, concerning the oldest age group (80 and over), men were at higher risk of victimization than women [5]. Previously, *The National Elder Abuse Incidence Study* [9] revealed that men were more likely to be the victims of abandonment (62.2% vs. 37.8% of women), whereas women were more likely to be victims of neglect (60.0% vs. 40% of men).

The book *Abuse of Older Men* by Kosberg [10], which represents an important expansion of the elder abuse literature [11], highlighted the existence in particular of Intimate Partner Violence (IPV) [12] and sexual abuse [13] across both genders. Additionally, some articles and media accounts from 1986 to 2007 have reported that older men were victims of economic exploitation [14].

In general, older men may need help with housing, legal matters and finances, and in particular they seem to be more vulnerable to financial exploitation by family members or others due to the possession of greater financial resources than older women [15]. In this respect, it is interesting to note a survey [16] regarding the opinion of General Practitioners (GPs), older carers, independent older adults and care-receiving older adults, on the seriousness of some potentially abusive scenarios. Findings showed that female GPs and female care-receivers rated financial abuse as being of lower importance than did male caregivers. A recent survey [17] found that 36% of older women and 35% of older men were victims of financial abuse, with males affected slightly more than females in total.

Concerning abuse in the context of personal and sexual relationships, Skirbekk and James [18] showed that older women experience a higher prevalence of most types of abuse than older men, except for physical violence (4.6% of men vs. 4.0% of women). Also data from the Office for National Statistics in England and Wales [19], concerning violent crime and sexual offences against men and women aged 16–59, showed that 38% of males were victims of domestic abuse. Moreover, a study in Stockholm [20] found that large numbers of men aged 18–64 had experienced complex violence, not the least of which was exposure to aggressive language and physical assault. Lifelong IPV is thus experienced by ageing men and women, including mutual abuse (e.g. emotional, sexual, and injuries) [21, 22]. A recent literature review on elder abuse in nursing homes [23] showed that male and female patients were victims of sexual abuse in those settings, although rates for females were higher than those for males.

Concerning emotional abuse, male victims were found to be more dependent on the perpetrator than female victims were (29% vs. 10%) [24]. Another study, investigating men with physical and cognitive disabilities [25], indicated that these men experienced abuse of worrying levels. A study by Dong and colleagues [26] found that depression was associated with increased risk of self-reported elder abuse in men and women. This risk could be modified by social support, which appears to have a stronger protective effect for men than for women, especially in terms of health benefits [27, 28].

According to Acierno [29], low social support seems to be a consistent, but modifiable, risk factor for all types of mistreatment in later life. In particular, with regard to people aged sixty years and over, Acierno found that 13.4% of males and 13.7% of females were victims of emotional mistreatment, whereas 2.4% of males and 1.5% of females were victims of physical abuse. The perpetrators were predominantly spouses and other family members. Older men were more likely than women to be emotionally (13% vs. 7%) and physically (8% vs. 1%) abused by strangers. Poole and Retschlin [30] also observed that the rates of physical and psychological mistreatment of older adults were similar with regard to gender.

Overall, that the phenomenon of the abuse of older men exists is to some extent supported by part of the available literature, although elder mistreatment is often reported as a typically female issue. Both women and men experience abuse and/or neglect in later life, especially when they show signs of disability and become dependent on others for help in practising their daily activities as a result [31]. In some cases (e.g. financial and physical abuse) men are victims more often than women [1, 4, 15, 18].

Moreover, some studies have shown that women can be perpetrators of such abuse, although the current literature often highlights them as acting in a caring, rather than violent, manner, whereas men are more likely to be reported as ‘the abusers’. In this respect, Straus [32] reviewed over 200 studies with general data on both abused men and women (of all ages), and found a ‘gender symmetry and mutuality’ among perpetrators, with about the same percentage of women and men having physically assaulted a partner. The *National Elder Abuse Incidence Study* [9] also revealed that, although 52.5% of the incidents involved male perpetrators, the remaining 47.5% of incidents were perpetrated by women. Archer [33] showed in particular that women often used acts of physical aggression towards their partners, although men inflicted injuries more frequently. A further study [34] found that females were abusive towards ageing relatives more often than males were (75.0% vs. 67%). Lövestad and Krantz [35] highlighted that both men and women were victims and perpetrators of physical violence. In particular, more men (11%) than women (8%) reported exposure to physical assault in the past year. According to Roberto and colleagues [36], most cases (73%) regarding IPV in later life dealt with violence against women by a male perpetrator. However, 15% of the episodes concerned violence against men by a female perpetrator. More recently [37] it was reported that men and women are perpetrators almost equally (52% of men vs. 48% of women).

Abuse of older men is thus real but still remains under-recognized and under-detected, under-investigated and under-theorized [38–40]. Male elder abuse also seems to be under-reported [41], due to victims experiencing feelings of shame and humiliation which can lead to reluctance towards both admitting such problems and seeking help [42]. Abused men may also avoid reporting victimization due to a fear of being ‘paid back’ for their own earlier acts of commission/omission towards their families [43].

Following this consideration, and taking into account the above mentioned findings, the scrutinization of the abuse of older men in Europe is very important for four main reasons: first, this worrying phenomenon exists, as it is highlighted also in the data from *Elder Abuse*: *A multinational prevalence survey* (ABUEL) [44]; second, unfortunately there are not many studies that confirm that older men are exposed to episodes of violence; third, few studies have specifically addressed the topic and its features from a comparative perspective within multi-cultural and multi-national contexts; and fourth, it is crucial to increase the awareness and reporting of male elder abuse in the population.

The aims of this study were thus: to describe the prevalence and characteristics of different types of abuse towards older men in the past 12 months in comparison to abuse of women; to examine differences in demographic, socio-economic and lifestyle variables between victimized and non-victimized men in all types of abuse; to identify factors associated with male elder abuse using a multilevel approach within the framework of an Ecological Model, in order to analyse the abuse of older men as an individual, family/community and societal question. We hypothesized that older men, similarly to older women, are also exposed to abuse and related risk factors. This exposure is associated with different dimensions: some of these are pertaining also to women (e.g. the ageing process), whereas some seem more specifically related to male gender (e.g. the greater vulnerability/dependency of older men).

## Materials and Methods

### Data Sources/Collection and Ethics Statement

The present study is based on data from the ABUEL Survey carried out between January and July 2009 [44, 45], which sought to investigate elder abuse in seven urban centres of seven European countries: Ancona (Italy), Athens (Greece), Granada (Spain), Kaunas (Lithuania), Ludwigsburg (Germany), Porto (Portugal) and Stockholm (Sweden). The data were collected cross-sectionally among community-dwelling elderly by face-to-face interviews, self-reporting or a combination of both approaches. Interviewers in each country were carefully instructed about ethical behaviour and the administration of the questionnaire. Written informed consent from participants, regarding their anonymity, rights and freedom to stop the interviews at any moment, was obtained prior to data collection. Ethical approval was sought and received in each participating country, from university, national, or regional ethics review boards, with the exception of Greece, where the fieldwork was carried out by the QED Company which is member of ESOMAR and provides global guidelines for ethics [45].

The full names of the other six ethics committees/institutional review boards were the following: Regional etisk kommittee vid Karolinska Institutet (Karolinska Institute, Regional Ethics Committee) in Sweden; Ethikkommission des Landes Baden-Wuerttemberg (Ethics Committee of the State of Baden-Wuerttemberg) in Germany; Comitato di Bioetica INRCA, Istituto Nazionale di Riposo e Cura per Anziani, Ancona (National Institute of Health and Science on Ageing, Bioethics Advisory Committee) in Italy; Kauno regioninio biomedicininiu tyrimu etikos komitetas (Kaunas Regional Research Ethics Committee) in Lithuania; Comité de Ética do Hospital de João, Porto (Ethics Committee of the John Hospital, Porto) in Portugal; Comité de Etica en Investigación de la Universidad de Granada (Research Ethics Committee, University of Granada) in Spain.

The final sample (gender- and age-stratified) included 4,467 persons (2,559 women) randomly selected (registry/census based) from the general population, except for Greece (where a sampling by random route of the elderly was obtained) and Portugal (where a cluster sampling method was used). The inclusion criteria across countries were: (a) women and men; (b) age 60–84 years; (c) not suffering from dementia or other cognitive impairments, assessed by means of the Mini-Cog test [46]; (d) having legal status (national citizenship or documented migrants status); (e) living in the community (homeowners or renters) or homes for elderly (e.g. sheltered housing). The sample size calculation was based on municipal censuses in each participating city, and on an expected abuse prevalence of 13% derived from a recent systematic review [47].

Assuming this prevalence rate, with a precision of 2.6%, a sample size of 633 individuals in each city was required, but considering the infinite population assumption a maximum of 656 individuals was allowed. The sample size was adapted to each city according to the population of individuals aged 60–84 years (representative and proportional to gender and age). Mean response rate was 45.2% across countries. More detailed description of materials and methods, sampling strategy and data collection, target population, cooperation, completion and response rates by country, are reported in a separate paper [48].

### Measures

The participants completed a standardized questionnaire with various validated instruments [45].

Violence was assessed with 52 questions based on the UK study on elder abuse [49] and the revised Conflict Tactics Scales (CTS2) [50]. The participants were asked if during the past year they had been exposed to at least one single episode/event of: psychological (11 items), physical (17 items), sexual (8 items) and financial abuse (9 items), including injuries (7 items). The acts of abuse may have occurred once, twice, three to five, six to ten, eleven to twenty, or over twenty times during the past year, or did not occur the past year. In addition, we assessed neglect (e.g. lack of help for routine housework) and data concerning the perpetrator’s main characteristics. For this study, the focus was on exposure to the above mentioned abuse types, excluding neglect.

Somatic symptoms were measured with the short version of the Giessen Complaint List (GBB) [51], consisting of 24 questions (graded 0–4, no symptoms to severely affected), with six questions in each of four types including: exhaustion (e.g. tiredness); gastrointestinal (e.g. nausea); musculoskeletal (e.g. pains in joints or limbs); and heart distress (e.g. heavy, rapid or irregular heart-throbbing). The total score amounts to 96, and the sub-total score in each symptom category ranges from 0–24. The higher the scores, the more one is affected. For this study, the focus was on the total score.

Depressive and anxiety symptoms were measured with the Hospital Anxiety and Depression Scale (HADS) [52]. This consists of 14 questions (graded 0–3), with seven questions about depression (e.g. I feel as if I am slowed down) and seven about anxiety (e.g. I get sudden feelings of panic). The total score for depression and anxiety is 21 each. A score of 0–7 corresponds to no cases, 8–10 to possible cases and 11–21 to probable cases. High scores correspond to high depression and anxiety levels. For this study, the focus was on the total score.

Healthcare use was measured as number of contacts with different types of healthcare staff (e.g. physician) and healthcare services (e.g. primary care). Additionally, we assessed the number of diseases (e.g. cardiovascular) currently suffered by the elderly. The questions were derived from the Stockholm County Council health survey [53].

Quality of life was measured with the WHO Quality of Life-Old (QoL) [54] consisting of 24 items (graded 1–5). The total score amounts to 100 and items are divided into 6 subscales, i.e. sensory abilities, autonomy, past, present/future activities, social participation, death/dying and intimacy. High scores correspond to high QoL (total/sub-scales). For this study the focus was on the total score.

Lifestyle variables were measured in terms of alcohol and cigarette use, religiosity and Body Mass Index (BMI). Alcohol was assessed with a modified version of Alcohol Use Disorders Identification Test [55] consisting of 5 items (e.g. do you drink alcohol?). A similar strategy was used for the assessment of cigarette use. Religiosity was assessed by the question: ‘Do you consider yourself a religious person?’ Finally, BMI was computed for each elderly person with the formula kg/m^2^. For this study the focus was on use of alcohol/cigarettes and religiosity in a ‘yes/no’ format.

Social support was measured with the Multidimensional Scale of Perceived Social Support (MSPSS) [56]. It consists of 12 questions (graded 1–7) and 3 sub-scales, i.e. support from family, significant other and friends. The possible range of each subtotal score is 4–28, and the possible range of total score (sum all responses) is 12–84. High scores correspond to high social support (sub-scales, total). For this study, the focus was on the total score.

Household size was assessed by the number of persons living with the interviewed person in the same home.

Various demographic and socio-economic variables were measured: gender, age (five-year groups: 60–64, 65–69, 70–74, 75–79, and 80–84); marital status (single, married/cohabiting, divorced/separated and widow/er); living situation (alone, only with partner/spouse, with partner/spouse/others, without partner/spouse but with others); habitation (living in a property owned by the respondent, in rented accommodation, or other e.g. housing for the elderly); education level (cannot read/write, without any degree of education, less than primary school, primary school/similar, secondary school/similar, university/similar, other); profession (managers/professionals/assistant professionals, clerical support/sales workers, skilled agricultural/forestry/fishery workers, assemblers/elementary occupations, housewife/husband, and armed forces); financial support (main source of income: work income, work pension, social/sick-leave/other pension benefits, partner/spouse income and other). Also, if the person was still working with a paid job this was noted. Finally, self-reported financial strain was investigated with the question: ‘How often are you worried about the daily expenses? (e.g. for buying food)’ and it was measured in a ‘no/sometimes/often/always’ format. The demographic and socio-economic variables were customized for each country, but were similar in content.

### The Ecological Model Approach for Elder Abuse

In order to explore factors related to elder abuse, a model reflecting multiple causes of the phenomenon was utilized, as recommended by the National Research Council (NRC) [57]. In this respect, the Ecological Model represents an interesting framework with which to explore potential risk factors and potential prevention strategies related to elder abuse [58, 59].

The model is drawn from previous conceptual frameworks concerning human ecological perspectives [60, 61]. The four-level Ecological Model considers mistreatment as the complex result of multiple factors influencing the relationship between individual and contextual factors, and it provides a holistic examination of elder abuse and useful insights for policy discussions [62], through an approach based on nested (rather than intersecting) systems. The model thus puts in evidence the importance of ‘levels or layers of thinking’ [63].

**Fig 1:**
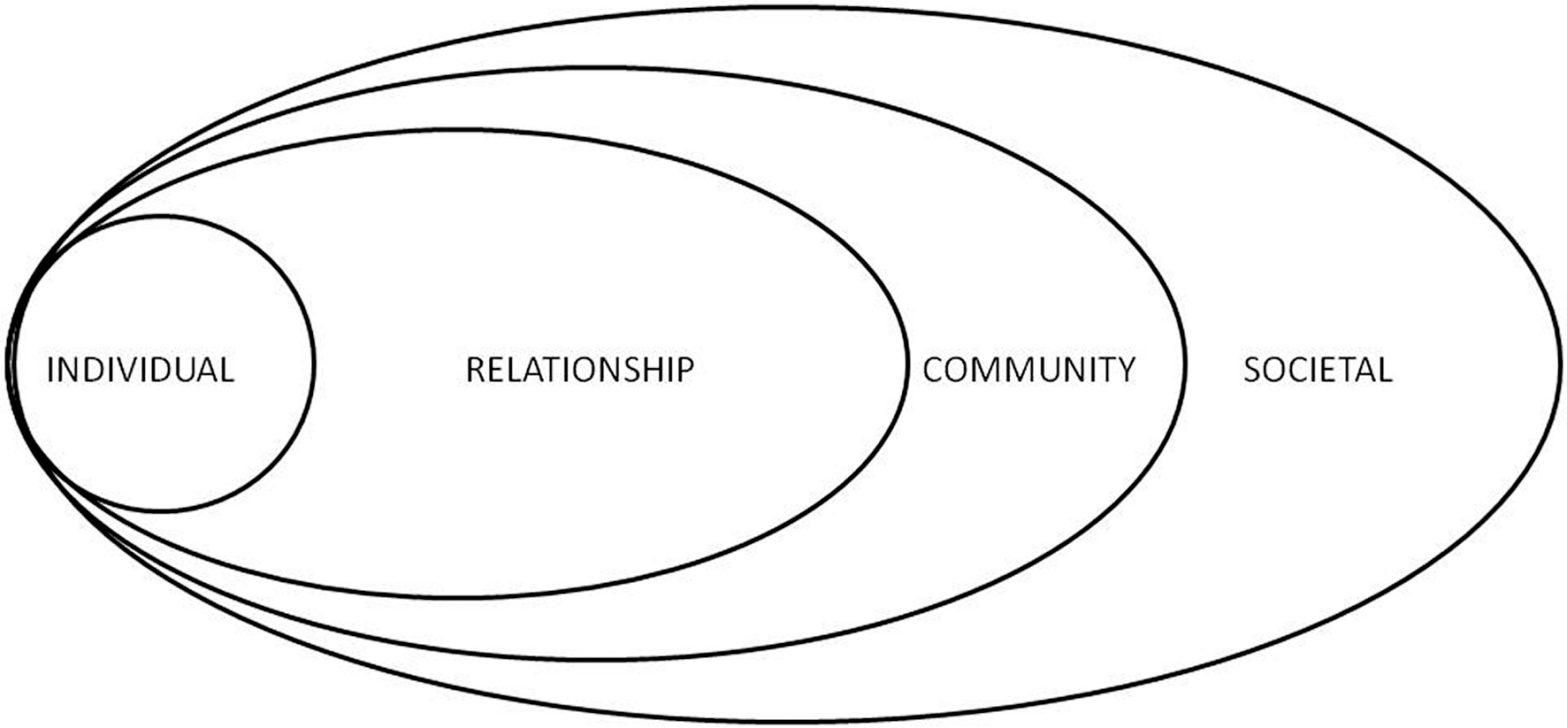
The Ecological Model. *Sources*: based on various references [58–61].

The model organizes various crucial aspects into groups and represents them in the outer rings of a series of concentric circles (see Fig 1). It allows the representation of interactions between macro-, meso- and micro-level factors, namely the following: individual (biological/personal factors, i.e. age, education, income, substance use, health); relationship (close relationships/interactions, i.e. the person’s closest social circle-peers, partners and family members); community (e.g. workplaces or other settings in which social relationships occur); social context in which abuse may be encouraged or inhibited (broad societal factors, social/cultural norms, i.e. health, economic, educational and social policies allowing socio-economic inequalities among individuals) [58].

The Ecological Model has been used by Edelson and Tolman [64] as a framework for exploring the phenomenon of female victims of elder abuse. In this paper we aimed to test the model for older abused men.

### Statistical Analyses

The bivariate relation between male victims/non-victims and categorical/ordinal variables (e.g. demographic and socio-economic characteristics) was analysed with the Chi-squared test. Associations between types of abuse and continuous variables (household size, BMI, healthcare services use, somatic symptoms, social support, depression, anxiety, and quality of life) were analysed by comparison of means value and T-tests. Furthermore, a multilevel logistic regression analysis, on stepwise Ecological Model, was used to examine male exposure to elder abuse and injury. In our analyses, the Ecological Model gives a visual depiction of the complex interplay between the individual, relationship, community and societal factors which relate to male elder abuse. To detect predictors indicative of increased probability of being abused, for each of the four levels a group of variables was associated, as a preparatory step towards providing the multilevel logistic regression analyses. Variables representing the ‘individual level’ were: age (included as continuous); educational level; proxies for income (i.e. habitation, still working and financial strain); proxies for health status (i.e. BMI, anxiety, depression and somatic symptoms); and lifestyle variables (i.e. smoking and alcohol use).

Concerning the individual variables, we excluded ‘financial support’ due to collinearity with ‘financial strain’. We included instead ‘financial strain’ due to its psychological aspect related to some fears/insecurities among the elderly, which often function as a precursor to possible incidents of abuse. As for the ‘relationship level’, variables included in this group were marital status and living situation. Concerning the relationship variables, we excluded ‘household size’ due to collinearity with ‘living situation’. We included ‘living situation’ because it provides more information on households apart from number of inhabitants. Regarding the ‘community level’, the selected variables were: profession, healthcare use, quality of life, perceived social support and religiosity. Finally the ‘societal level’ was described by country (Italy, Greece, Spain, Lithuania, Germany, Portugal and Sweden).

Given the different levels of data (micro-, meso- and macro-level factors, respectively at the individual, relationship/community and country levels), the statistical model had to take into account the existence of a clustered structure [65] since each country has a specific cultural background and people living in the same area tend to experience similarities. As the dependent variable is dichotomous (whether or not an individual is the victim of abuse), it was necessary to estimate a binary choice model, therefore multilevel logistic regression was selected in order to allow the decomposition of total variability into a primary level (subject-related variability) and a secondary level (country variability) [66].

In the four regressions of the Ecological Model, the geographical area of residence was included as random-effect parameter, whereas covariates were included with a forward stepwise procedure according to the levels of the Ecological Model we took into consideration. Regression 1 analyzed the crude between-country variance in older male abuse as a random effect, i.e. the ‘societal level’; Regression 2 included the variables comprehended in the ‘individual level’; Regression 3 added those pertaining to the ‘relationship level’; Regression 4 included those of the ‘community level’.

The significance level for all analyses was set at *p<0*.*05*. The relationship between the country and the total variability (intra-class correlation, ICC) was used to calculate the weight of the variability between clusters. Odds ratios (OR), Confidence Intervals (CI) and *p*-values for each variable are presented. A Likelihood Ratio test (LR) versus logistic regression was performed to support the choice of a multilevel approach, whereas the validity of the regressions was assessed by means of the diagnostic Wald-test of joint ‘0’ coefficients. The statistical packages SPSS 17.1 and STATA 11.2 were used to carry out the analyses.

## Results

### Descriptive Statistics

Table 1 provides a descriptive summary of the demographic and socio-economic characteristics of the sample. Of the responses (n = 4,467), 57.3% were women. Among participants, 6% were single and 65% married or cohabiting, whereas 49.6% lived only with a partner/spouse and 24.2% lived alone. Moreover, 76% were homeowners, 39.9% had secondary/intermediate education, 27.6% were managers/professionals and 27.5% were clerical support/sale workers. As for professional/economic condition, 65.9% lived on a work pension, 17.6% were still working and 64% declared that they experienced financial strain. Finally, 12% reported that they smoked, 64% that they drank alcohol, and 86% considered themselves to be religious. More detailed descriptions of the study sample and demographic/socio-economic aspects are presented in separate papers [45, 67, 68].

**Table 1:** Demographic and socio-economic characteristics of the sample.

	Total (n = 4467)	
Variables	N	%
*Country*		
Germany	648	14.5
Greece	643	14.4
Italy	628	14.1
Lithuania	630	14.1
Portugal	656	14.7
Spain	636	14.2
Sweden	626	14.0
*Age group (years)*		
60–64	1124	25.2
65–69	1088	24.4
70–74	961	21.5
75–79	749	16.8
80–84	545	12.2
*Gender*		
Female	2559	57.3
Male	1908	42.7
*Marital status*		
Single	270	6.0
Married/cohabiting	2903	65.0
Divorced/separated	343	7.7
Widow/er	950	21.3
*Living situation*		
Alone	1078	24.2
Only partner/spouse	2208	49.6
Partner/spouse//others ^a^	706	15.9
Without partner/spouse—with others ^a^	457	10.3
*Habitation*		
Own	3392	76.0
Rental	930	20.8
Other ^b^	143	3.2
*Education*		
Cannot read/write	136	3.0
Without any degree	187	4.2
Less than primary school	338	7.6
Primary school/similar	1092	24.5
Secondary school/similar	1782	39.9
University/similar	855	19.2
Other ^c^	73	1.6
*Profession*		
Managers/professionals/assistant professionals	1217	27.6
Clerical support/sale workers	1214	27.5
Skilled agricultural/forestry/fishery workers	707	16.0
Assemblers/elementary occupations	570	12.9
Housewife/husband	656	14.9
Armed forces	45	1.0
*Main source of financial support*		
Work	542	12.1
Work pensions	2939	65.9
Social/sick-leave/other pension benefits ^d^	243	5.4
Partner/spouse income	627	14.1
Other ^e^	110	2.5
*Still working (paid work)*		
No	3518	82.4
Yes	751	17.6
*Financial strain* (*are you worried about daily expenses*?*)*		
No	1605	36.0
Yes	2857	64.0
*Smoking*		
No	3927	88.0
Yes	536	12.0
*Drinking*		
No	1598	35.8
Yes	2866	64.2
*Are you religious*?		
No	626	14.1
Yes	3813	85.9

^a^ = e.g. daughter;

^b^ = e.g. housing for elderly;

^c^ = e.g. art school;

^d^ = e.g. sick pension;

^e^ = e.g. own capital.

#### Internal Reliability of Exposure Variables

Reliability, considered as internal consistency of exposure variables across countries included in the study, was assessed using the Cronbach's α statistic [45]. Cronbach’s α for violence was: for psychological 0.85, for physical 0.80, for sexual 0.76, for financial 0.64, and for injuries 0.70. Moreover, Cronbach’s α was: for somatic symptoms 0.92, for anxiety 0.81, and for depression 0.80. Finally, Cronbach’s α was 0.92 for both QoL and total social support.

### Bivariate Analyses

#### Types of elder abuse by country and gender

As shown in Table 2, for the entire sample (across countries) and considering any type of abuse, 22.8% of the victims were male and 20.6% female. Furthermore, men had more often been abused than women specifically concerning psychological (20% vs. 18.9%), physical (2.8% vs. 2.6%), and financial abuse (4.1% vs. 3.7%). Notably, men were more abused than women in Italy (18.8% vs. 9.3%) and Sweden (36.9% vs. 25.8%). In Germany the total percentages of male and female abuse were quite similar (respectively, 30.2% and 30.6%). With regard to the other countries, were females more abused than males, particularly in Greece.

**Table 2:** Prevalence (%) of abuse and injury during the past 12 months by country and gender.

Country/gender	Psychological ^a^	Physical ^b^	Sexual ^c^	Financial ^d^	Injury ^e^	Any
*Germany* (n = 648)	27.1	3.3	0.9	3.6	0.4	30.4
Female (n = 343)	26.8	2.8	1.5	3.5	0.5	30.6
Male (n = 305)	27.5	4.0	0.0	3.8	0.3	30.2
*Greece* (n = 643)	13.2	3.4	1.5	4.0	1.1	15.7
Female (n = 356)	14.7	4.6	2.5	4.8	1.7	18.3
Male (n = 287)	11.3	2.0	0.3	3.1	0.3	12.6
*Italy* (n = 628)	10.4	1.0	0.5	2.7	0.0	12.7
Female (n = 358)	6.9	1.2	0.6	2.0	0.0	9.3
Male (n = 270)	16.5	0.7	0.4	3.8	0.0	18.8
*Lithuania* (n = 630)	24.6	3.8	0.3	2.8	1.5	26.2
Female (n = 405)	25.1	4.1	0.4	2.4	2.0	26.4
Male (n = 225)	23.7	3.1	0.0	3.5	0.7	25.9
*Portugal* (n = 656)	21.9	2.1	1.3	7.8	0.7	27.6
Female (n = 400)	25.4	2.0	1.6	6.6	1.2	29.6
Male (n = 256)	16.6	2.2	0.8	9.6	0.0	24.6
*Spain* (n = 636)	11.5	1.4	0.3	4.8	0.5	14.5
Female (n = 364)	12.8	1.4	0.2	5.5	0.3	15.9
Male (n = 272)	9.7	1.4	0.4	3.9	0.9	12.5
*Sweden* (n = 626)	29.7	4.0	0.5	1.8	0.6	30.8
Female (n = 333)	24.9	2.3	0.6	1.7	0.6	25.8
Male (n = 293)	35.6	6.0	0.4	2.0	0.7	36.9
*Total* (n = 4467)	19.4	2.7	0.7	3.8	0.7	22.1
Female (n = 2559)	18.9	2.6	1.0	3.7	0.9	20.6
Male (n = 1908)	20.0	2.8	0.3	4.1	0.4	22.8

^a^ = e.g. undermined or belittled what you do;

^b^ = e.g. kicked you;

^c^ = e.g. touched you in a sexual way against your will;

^d^ = e.g. tried to make you give money, possessions or property;

^e^ = e.g. you passed out from being hit on the head.

#### Any type of abuse and demographic/socio-economic characteristics of male victims and non-victims

As shown in Table 3, we found significant geographical differences between the two groups of males (*p<0*.*001*), with victims most prevalent in Sweden (24.4%) and Germany (19.6%), whereas non-victims were prevalent in Greece (16.9%) and Spain (16.3%). Other significant differences (*p<0*.*01*) were found concerning age. Victims were concentrated in the age groups 60–64 and 70–74 years (respectively, 29.4% and 26.4%), and non-victims were on the whole older than victims (28.4% against 21.2% in the age group 75–84). As for living situation (*p<0*.*01*), victims were more likely to live alone or only with a spouse/partner (14.8% and 65.7%) than non-victims (12.5% and 59.7%). Victims vs. non-victims scored lower on financial strain (52.6% vs. 59.8%, *p<0*.*01*) and were educated to higher levels (*p<0*.*01*), with university degrees (29.4% vs. 21.3%). Minor significant differences (*p<0*.*05*) were found concerning greater rates among victims than non-victims, as for being divorced/separated, not religious, living in rented accommodation, still working, managers/professionals and assemblers/with elementary occupations.

**Table 3:** Abuse and injury (any type) of men by demographic/socio-economic and lifestyle variables. (Did any kind of violence occur in the last year?), %.

Variables	Male non-victims (n. 1465)	Male victims (n. 443)	Men total (n. 1908)
Country***			
Germany	14.9	19.6	16.0
Greece	16.9	8.8	15.0
Italy	15.0	11.5	14.2
Lithuania	11.1	14.0	11.8
Portugal	13.2	14.2	13.4
Spain	16.3	7.5	14.3
Sweden	12.6	24.4	15.4
Age group (years)**			
60–64	25.7	29.4	26.5
65–69	26.2	23.0	25.5
70–74	19.7	26.4	21.2
75–79	17.3	11.7	16.0
80–84	11.1	9.5	10.7
Marital Status*			
Single	4.6	3.8	4.5
Married/Cohabiting	80.5	80.8	80.6
Divorced/Separated	4.5	7.7	5.2
Widower	10.4	7.7	9.8
Living situation**			
Alone	12.5	14.8	13.1
Only spouse/partner	59.7	65.7	61.1
Spouse/partner/other ^a^	22.1	16.4	20.8
Without partner/spouse—with others ^a^	5.7	3.2	5.1
Habitation*			
Own	79.9	73.8	78.4
Rental	17.8	24.0	19.3
Other ^b^	2.3	2.3	2.3
Education**			
Cannot read/write	2.1	0.9	1.8
Without any degree	3.2	2.3	3.0
Less than primary school	6.9	2.9	6.0
Primary school/similar	23.4	22.4	23.2
Secondary school/similar	41.6	40.6	41.3
University/similar	21.3	29.4	23.1
Other ^c^	1.5	1.6	1.5
Profession*			
Managers/professionals/assistant professionals	33.6	41.1	35.3
Clerical support/sale workers	26.1	20.2	24.7
Skilled agricultural/forestry/fishery workers	23.0	21.6	22.7
Assemblers/elementary occupations	14.2	15.3	14.5
Husband	0.6	0.2	0.5
Armed forces	2.6	1.6	2.3
*Main source of financial support*			
Work	14.7	19.4	15.8
Work pension	78.4	72.7	77.1
Social/sick-leave/other pension benefits ^d^	4.6	5.4	4.8
Partner/spouse income	0.6	0.9	0.7
Other ^e^	1.7	1.6	1.7
Still working (paid work)*			
No	79.8	74.5	78.6
Yes	20.2	25.5	21.4
*Financial strain* (*are you worried about daily expenses*?*)* **			
No	40.3	47.4	41.9
Yes	59.8	52.6	58.1
*Smoking*			
No	83.0	85.5	83.6
Yes	17.0	14.5	16.4
*Drinking*			
No	24.0	20.1	23.1
Yes	76.0	79.9	76.9
*Are you religious*?*			
No	19.9	24.6	21.0
Yes	80.1	75.4	79.0

^a^ = e.g. daughter;

^b^ = e.g. housing for elderly;

^c^ = e.g. art school;

^d^ = e.g. sick pension;

^e^ = e.g. own capital;

* p<0.*05;*

** p<0.*01;*

*** p<0.*001*.

#### Abuse and injury of men by household size, BMI, healthcare use and somatic symptoms

As shown in Table 4, psychological abuse victims scored higher than non-victims on somatic symptoms (14.6 vs. 11.5, *p<0*.*001*) and healthcare use (as expressed by frequency of healthcare contacts) (17.2 vs. 16.8, *p<0*.*05*), and lower on household size (2.1 vs. 2.3, *p<0*.*05*). Victims of physical abuse also scored lower than non-victims on household size (1.9 vs. 2.3, *p<0*.*05*). Victims of financial abuse scored significantly higher than non-victims on somatic symptoms (16.4 vs. 12.0, *p<0*.*01*).

**Table 4:** Abuse and injury of men by household size, BMI, healthcare use and somatic symptoms.

Variables	Household size ^a^				BMI ^b^				Healthcare use ^c^				Somatic symptoms ^d^			
	n	Mean	s.d.	*p*^l^	n	Mean	s.d.	*p*^l^	n	Mean	s.d.	*p*^l^	n	Mean	s.d.	*p*^l^
*Psychological* ^e^				*<0*.*05*				*= 0*.*195*				*<0*.*05*				*<0*.*001*
No	1517	2.3	1.0		1478	26.8	3.8		1490	16.8	3.0		1521	11.5	11.6	
Yes	385	2.1	0.8		383	26.5	3.8		382	17.2	2.4		387	14.6	13.3	
*Physical* ^f^				*<0*.*05*				*= 0*.*738*				*= 0*.*160*				*= 0*.*671*
No	1849	2.3	1.0		1809	26.7	3.7		1819	16.9	2.9		1855	12.1	11.9	
Yes	53	1.9	0.6		52	26.5	4.9		53	17.4	2.8		53	12.8	15.6	
*Sexual* ^g^				*= 0*.*826*				*= 0*.*091*				*= 0*.*101*				*= 0*.*134*
No	1896	2.2	1.0		1855	26.7	3.8		1866	16.9	2.9		1902	12.1	12.0	
Yes	6	2.3	1.2		6	24.1	5.1		6	18.8	2.5		6	19.5	10.9	
*Financial* ^h^				*= 0*.*064*				*= 0*.*276*				*= 0*.*094*				*<0*.*01*
No	1821	2.3	1.0		1783	26.7	3.8		1792	16.9	2.9		1827	12.0	11.8	
Yes	81	2.0	0.8		78	27.2	3.6		80	17,4	2,6		81	16,4	15,3	
*Injury* ^i^				*= 0*.*071*				*= 0*.*124*				*= 0*.*469*				*= 0*.*089*
No	1894	2,2	1,0		1853	26.7	3.8		1864	16.9	2.9		1900	12.1	12.0	
Yes	8	1.6	0.7		8	24.7	2.2		8	17.6	2.3		8	19.4	15.5	

^a^ = number of persons in the household;

^b^ = body mass index;

^c^ = number of healthcare visits;

^d^ = GBB-24, 0–96;

^e^ = e.g. undermined or belittled what you do;

^f^ = e.g. kicked you;

^g^ = e.g. touched you in a sexual way against your will;

^h^ = e.g. tried to make you give money, possessions or property;

^i^ = e.g. you passed out from being hit on the head;

^l^ = *p<0*.*05*.

#### Abuse and injury of men by social support, quality of life, depressive and anxiety symptoms

As shown in Table 5, male victims of sexual abuse, compared with non-victims, scored higher on anxiety symptoms (7.7 vs. 4.0, *p<0*.*05*). Moreover, victims of psychological abuse reported significantly higher scores than non-victims on depressive symptoms (5.4 vs. 4.4, *p<0*.*001*) and lower scores on quality of life (68.0 vs. 70.0, *p<0*.*05*). Finally, men exposed to psychological, financial abuse and injuries reported lower scores in total perceived social support than their counterparts, and this was particularly evident among those who sustained injuries (52.3 vs. 68.2, *p<0*.*001*).

**Table 5:** Abuse and injury of men by social support, quality of life, depressive and anxiety symptoms.

Variables	Social Support ^a^				Depressive symptoms ^b^				Anxiety symptoms ^c^				Quality of life ^d^			
	n	Mean	s.d.	*p*^l^	n	Mean	s.d.	*p*^l^	n	Mean	s.d.	*p*^l^	n	Mean	s.d.	*p*^l^
*Psychological* ^e^				*<0*.*001*				*<0*.*001*				*<0*.*001*				*<0*.*05*
No	1487	69.1	13.2		1500	4.4	3.8		1502	3.8	3.4		1414	70.0	14.4	
Yes	378	64.6	15.5		384	5.4	3.7		386	4.9	3.8		370	68.0	13.8	
*Physical* ^f^				*= 0*.*373*				*= 0*.*262*				*<0*.*05*				*= 0*.*807*
No	1816	68.2	13.7		1831	4.6	3.8		1836	4.0	3.5		1733	69.6	14.3	
Yes	49	66.4	16.4		53	5.2	4.1		52	5.0	4.1		51	69.1	13.4	
*Sexual* ^g^				*= 0*.*861*				*= 0*.*769*				*<0*.*05*				*= 0*.*451*
No	1859	68.2	13.8		1878	4.6	3.8		1882	4.0	3.5		1779	69.6	14.3	
Yes	6	69.2	20.7		6	4.2	3.1		6	7.7	3.7		5	74.4	13.7	
*Financial* ^h^				*<0*.*05*				*= 0*.*336*				*<0*.*01*				*= 0*.*822*
No	1785	68.3	13.6		1804	4.6	3.7		1807	3.9	3.5		1705	69.6	14.2	
Yes	80	64.7	17.0		80	5.0	4.1		81	5.1	4.8		79	69.2	15.2	
*Injury* ^i^				*<0*.*01*				*= 0*.*185*				*<0*.*05*				*= 0*.*251*
No	1859	68.2	13.7		1876	4.6	3.8		1880	4.0	3.5		1776	69.6	14.3	
Yes	6	52.3	29.6		8	6.4	4.1		8	6.6	4.5		8	63.8	18.2	

^a^ = MSPSS, 12–84;

^bc^ = HADS, 0–21;

^d^ = WHOQOL-OLD, 0–100;

^e^ = e.g. undermined or belittled what you do;

^f^ = e.g. kicked you;

^g^ = e.g. touched you in a sexual way against your will;

^h^ = e.g. tried to make you give money, possessions or property;

^i^ = e.g. you passed out from being hit on the head;

^l^ = *p<0*.*05*.

### Multivariate Analyses

#### Factors associated with male abuse: a multilevel approach in the framework of an Ecological Model

Results of the multilevel logistic regression analyses for each level are presented in Table 6. The intercept-only model (Regression 1) revealed that a certain amount of variation in abuse prevalence between countries exists since the ICC indicated that 6% of the total variance in male abuse could be accounted for by country-level effects. We observed that the ICC remained the same in Regression 2, and even including the ‘individual level’ variables, the impact of the ‘societal level’ in determining the probability of being abused was unvaried. Once ‘relationship’ and ‘community’ levels are taken into account, a decreasing trend in the ICC in regressions 3 and 4 is observed, reaching the 3% of the total variance.

**Table 6:** Multilevel Logistic Regression Analyses (on stepwise Ecological Model) of male exposure to elder abuse and injury*.

Levels	Effects	Regression 1 ^a^			Regression 2 ^b^			Regression 3 ^c^			Regression 4 ^d^		
		n = 1908			n = 1808			n = 1803			n = 1615		
	Fixed	OR	*p* ^i^	[95% Cl]	OR	*p* ^i^	[95% Cl]	OR	*p* ^i^	[95% Cl]	OR	*p* ^i^	[95% Cl]
**Individual**	*Age*				0.98	0.06	0.96–1.00	0.98	0.07	0.96–1.00	0.98	0.03	0.96–1.00
	*Education* (*ref*. *Low*) ^e^												
	Middle				1.17	0.29	0.88–1.56	1.16	0.31	0.87–1.55	1.23	0.22	0.89–1.70
	High				1.46	0.02	1.05–2.02	1.47	0.02	1.06–2.03	1.56	0.05	0.99–2.46
	*Habitation* (*ref*. *Own*) ^f^												
	Rental				1.36	0.04	1.01–1.82	1.39	0.03	1.03–1.87	1.38	0.05	1.00–1.90
	*Still working* (*ref*. *No*)												
	Yes				1.12	0.46	0.83–1.51	1.14	0.39	0.84–1.54	1.07	0.66	0.78–1.48
	*Financial strain* (*ref*. *No*)												
	Yes				0.77	0.04	0.59–0.99	0.77	0.05	0.59–1.00	0.73	0.02	0.55–0.96
	*Smoking* (*ref*. *No*)												
	Yes				0.87	0.41	0.63–1.21	0.90	0.51	0.64–1.24	0.85	0.37	0.60–1.21
	*Drinking* (*ref*. *No*)												
	Yes				0.92	0.60	0.67–1.25	0.91	0.53	0.66–1.24	1.03	0.88	0.73–1.43
	*BMI*				1.00	0.98	0.97–1.03	1.00	0.84	0.97–1.03	1.00	0.84	0.97–1.04
	*Somatic symptoms (GBB)*				1.02	0.00	1.01–1.03	1.02	0.00	1.01–1.03	1.02	0.00	1.01–1.03
	*Depressive symptoms (HADS)*				1.03	0.15	0.99–1.07	1.03	0.12	0.99–1.08	1.02	0.43	0.97–1.07
	*Anxiety symptoms (HADS)*				1.06	0.00	1.02–1.10	1.06	0.01	1.02–1.10	1.06	0.00	1.02–1.11
**Relational**	*Marital status* (*ref*. *Single*) ^g^												
	Married/cohabiting							1.14	0.73	0.55–2.34	1.45	0.37	0.64–3.29
	*Living situation* (*ref*. *Alone*)												
	Only partner/spouse							1.05	0.90	0.48–2.27	1.02	0.96	0.42–2.49
	Partner/spouse/others							0.88	0.76	0.39–1.98	0.84	0.71	0.33–2.11
	Without partner-with others							0.81	0.54	0.41–1.59	0.96	0.92	0.46–2.00
**Community**	*Profession* (*ref*. *Blue-collar*) ^h^												
	LowWhite-collar										0.69	0.03	0.49–0.97
	Middle/High White-collar										0.80	0.26	0.54–1.18
	*Quality of Life (QoL)*										1.01	0.24	0.99–1.02
	*Social support (MSPSS)*										0.98	0.00	0.97–0.99
	*Are you religious*? (*ref*. *No*)												
	Yes										0.99	0.94	0.72–1.35
	*Healthcare use*										1.03	0.23	0.98–1.07
	**Random**												
**Societal**	*Country*												
	Variance	0.21	-	0.07–0.68	0.20	-	0.06–0.70	0.18	-	0.05–0.65	0.11	-	0.02–0.48
	ICC	0.06	-	-	0.06	-	-	0.05	-	-	0.03	-	-
	LR test *p value*	0.00	-	-	0.00	-	-	0.00	-	-	0.00	-	-

* *Dependent/dichotomous variable*: victim of abuse: yes/no;

^a^ = crude between-country variance in older male abuse as a random effect (Societal level);

^b^ = included the variables comprehended in the Individual Level;

^c^ = added Relationship Level variables;

^d^ = included also Community Level variables;

^e^ = education recoded as Low (cannot read nor write; without any degree; less than primary school; primary school/similar), medium (secondary education, similar e.g. middle high school, other) and high (university/similar);

^f =^ habitation recoded as own and rented place, answers included in ‘other’ were distributed inside the previous categories;

^g =^ marital status recoded as single (single; divorced/separated; widower) and married/cohabiting;

^h =^ profession recoded as blue-collar workers (skilled agricultural forestry and fishery workers; assemblers/elementary occupations; husbands); low white-collar workers (clerical support workers and sales work) and middle/high white-collar workers (managers, professionals, assistant professionals, armed forces);

^i =^ *p<0*.*05*.

Regarding fixed effects, in Regression 2 we found that some individual variables played a statistically significant role in predicting the probability of being abused. In particular older men educated to higher levels were more likely to report abuse than those educated to lower levels (46% more in Regression 2; 47% more in Regression 3; 56% more in Regression 4). A similar result was observed for those living in rented accommodation compared to homeowners (almost 40% more in the three regressions), whereas for older men who were worried about daily expenses the probability of being abused decreased by 23% in regressions 2 and 3, and by 27% in Regression 4. In addition, when somatic and anxiety symptoms increased, the probability of being abused increased too.

These results remained unchanged when the ‘relationship level’ variables were included (Regression 3) for both significance and odds ratios, whereas marital status and living situation did not seem to influence the probability of being abused. Once ‘community level’ variables were included in Regression 4, further effects were observed. In addition to individual variables already significant in the previous regressions, age appeared to gain statistical significance, namely increasing age decreased the probability of being abused. As for the ‘community level’ variables, profession and social support predicted the probability of being abused. Low white-collar workers were 30% less abused than blue-collar workers, and with the increase of social support the probability of being abused decreased too.

## Discussion

The aim of our discussion is to process significant findings from the multivariate analyses, trying to provide an overall picture of the phenomenon within the framework of the Ecological Model, which is a useful approach to integrating micro-, meso- and macro-processes [69, 70]. We started from the following assumptions: elder abuse is the product of multiple levels of influence on behaviour; thus it results from the interaction of personal, relationship, cultural and environmental factors; and as such no single dimension can explain in depth this sensitive and complex phenomenon [7]. Our results suggest indeed that individual, community, and societal factors are associated with male elder abuse. The associations with relational-level factors were not statistically significant, i.e. when considered in a multivariate analysis, marital status and living situation did not seem to influence the probability of older men being abused. It is also important to clarify that in the discussion we propose explanations of results which are male specific but also further explanations related to elder abuse in general, thus concerning both men and women as victims of mistreatment. In the final analysis, the reasons leading to male or female elder abuse seem indeed more similar than different with regard to some aspects, and generally they relate to older age conditions and to violence against vulnerable people [71].

### Societal Level

#### Country

We found some variation in abuse prevalence between countries, and this could be accounted for by societal-level effects. This finding indicates that older male respondents from the same country/area/neighbourhood are subject to common geographical and societal influences/boundaries, and thus they are more similar to each other in relation to their exposure to abuse than they are to people from other areas. There are indeed variations among men across countries in terms of norms influencing behaviours, attitudes and relationships with women in society, and within family and marriage [43].

The idea of abuse as a ‘contextual phenomenon’ [72] provides further evidence for the relevance of different historical, geographical, cultural, political, and economic contexts for understanding the phenomenon itself [73]. This seems also to be the case for social support, an aspect that is tightly related to elder abuse as an overall protective factor [45, 74]. The presence of supportive social networks is indeed bound to cross-national/cultural variations [75, 76]; in particular a strong ‘family-centred’ cultural tradition is evident in Mediterranean countries, whereas greater support from non-family networks is reported in non-Mediterranean countries [77]. In other words, taking into consideration the issue of cultural diversity related to elder abuse (which affects elder abuse in general and not only with regard to male victims) represents a crucial issue of complexity for understanding the phenomenon [78]. In particular it should be considered that there are some cultural and social norms (e.g. ageism, sexism, marginalization and a culture of violence) which are tolerant and supportive of violence, and which are differently perceived and diffused across countries.

Also the mass media can contribute to the dissemination of attitudes and beliefs, providing a kind of ‘normalization’ of violence, thus resulting in increased manifestation of the phenomenon in some areas [7]. Furthermore, different economic and social policies can generate economic and social inequalities within societies, and this in turn could create contexts in which tension results in episodes of elder abuse, especially when older people depend financially on others [7].

### Individual Level

#### Age

In our study, among personal and individual level risk factors for victimization, increasing age reduced the probability of being abused. This finding seems in contrast with previous literature showing in general that the risk of maltreatment increased with age [5, 79–81], and in particular among people aged 74 years and older [7]. Older people in later life become frail and vulnerable due to bad health, and become dependent on others to help them undertake quotidian activities. These dependencies could in turn expose the elderly to episodes of violence. Our findings highlighting older men as less abused than younger men could reflect the fact that they had less capacity to report episodes of violence. When the elderly are abused they often feel a sense of shame, humiliation and powerlessness about ‘what happened’, and this can lead older men particularly to feel resigned and to them denying any mistreatment having taken place [15, 42]. Elderly men could thus prefer to ignore the incident and to avoid any reporting or reaction, thus highlighting their extreme fragility and vulnerability in these circumstances.

The social construction of manhood and cultural male stoicism are additional crucial factors for understanding the reasons that prevent older abused men from reporting and seeking support [82]. Kosberg [43] in particular reported evidence on the existence of a ‘male gender-role socialization’ in different cultures/countries. Men, from childhood, are educated and socialized to be stoic, strong and independent.

Older age in general seems to be linked also to a lower awareness of the phenomenon of abuse. In this respect Daskalopoulos and Borrelli [83] indicated that in general the older the individuals were, the less concerned they were with physical and psychological neglect. Thus to be/feel aged seems to act as self-justification for eventual episodes of experienced mistreatment [84], whereas an increased understanding of elder abuse could help in the recognition and reporting of violent behaviours [85]. Furthermore, Soares and colleagues [20] also found that male victims of violence (in their lifetime or in the past 12 months) were more often younger, and they suggested that younger males might be more subjected to abusive behaviour due to a lack of experience and ability to cope with or avoid violent situations.

#### Education

The education level of male victims also seems to play a role in abuse. In our study older men educated to higher levels were more often victims than their counterparts who were educated to lower-levels. This is contrary to results reported in previous studies, which indicate an opposite situation (not only for male victims), namely that risk factors for mistreatment include low levels of education [18, 26, 86]. In particular, educational level emerged as a protective factor for verbal abuse [87]. A further recent study [88] confirmed the negative link between education and reporting of episodes of abuse, and five to nine years of schooling was associated with 83% decreased odds of victimization.

However, a reverse pattern may be possible, as our study indicates. The higher level of education may indeed be significantly associated with the odds of reporting abuse, with more educated older men being more likely to perceive, recognize and report the possibility of being victims of mistreatment. This unusual positive association between educational background and elder abuse incidence has been also found in other studies [89, 90]. In particular, elderly victims of financial exploitation emerged as more educated, informed, and socially active [91], and in general being older and male, and with a lower level of education (besides being unmarried and with no involvement in social activities), were all factors linked to a lack of awareness and knowledge of abuse [92]. Furthermore, some recent findings [93] reported that respondents with a lower level of education were more likely to show a ‘vague’ or absent perception of the meaning of ‘elder abuse’. Higher education level thus, rather than representing a real risk factor, is more likely to reflect reporting bias, and a higher educational level can increase awareness and willingness to acknowledge and report interpersonal violence. More research is needed in order to understand whether higher education also means a higher propensity to refer to episodes of mistreatment or conversely if it is a real risk factor for the incidence of the phenomenon [94].

#### Habitation

Men living in a rented accommodation were more likely to refer to episodes of abuse than homeowners. This association seems to be related to less financial security and consequently more economic dependence of the victims on others, with older men in particular often being dependent on relatives for various needs (housing, legal matters and finances) [15]. Increasing financial dependency of older people is indeed a relevant risk factor for victims of abuse. Various studies found low income and poverty to be associated with elder abuse [95, 96]. Furthermore, stress may occur and provide episodes of abuse when financial resources are not sufficient to meet the needs of an ageing adult [94]. However, some studies report an opposite context, with home-owning older people being more likely to be exploited, due to valuable and visible property [97, 98].

#### Financial strain

Contrary to our expectation, we found that worries about daily expenses (financial strain) were associated with a decreased probability of being abused. This finding contrasts with the results mentioned above (i.e. rented accommodation/low income and higher risk of abuse). However we must consider that financial strain in our study was recorded as a perception and thus some bias could be possible, whereas the condition of owning or not owning a home represents a concrete situation. Moreover, one may reasonably speculate, for example, that a person without any disposable income, and thus reporting feelings of financial strain, is less vulnerable to financial exploitation. Kosberg [43] in particular observed that when an older man is financially dependent upon the abuser (family/non-family caregiver), he may also think that abuse episodes in general are due to the condition of economic dependency itself, and consequently he may not report to be a victim of any mistreatment.

The opposite context is also possible. Worrying about daily expenses could represent an indirect signal of economic abuse, i.e. a person may have disposable income and report financial strain as the victim of financial exploitation, but he may not refer explicitly to the situation as abusive. Some evidence suggests that older men usually have greater economic resources than older women and therefore they may be more exposed to financial abuse [15], especially when aged 80 years and over [44].

In this respect three scenarios are possible. Older men may depend on their spouse/partner or children for domestic activities (e.g. cleaning and cooking), and thus they may hesitate to report experiences of (e.g. financial) mistreatment, even if this results in continuing to live in an abusive relationship [99]. The older person may be therefore aware he is the victim of financial exploitation, and may refer to financial strain, but tacitly consents to be abused, especially if relatives are the perpetrators. In this case it is very difficult to detect and tackle the episode of mistreatment [100]. Otherwise, older men could be worried about expenses but are not aware they are victims of financial abuse, if for instance the relative perpetrator (who may, in this scenario, be a relative) takes money from their pension or from bank account without permission [91, 101, 102]. When older persons receive negative but misleading information on their economic situation, this indeed influences their perception of their real financial context. They think they are poor, but they could be victims of financial abuse. It should be highlighted that older men typically have little knowledge of how to change or freeze a bank account, and also in general with regard to the safeguarding of their economic assets [15]. Besides the two above mentioned scenarios regarding reporting financial strain but not referring to or not perceiving economic abuse, a further context is possible: the elder male refers to worry about daily expenses but doesn’t admit to being victim of financial exploitation because he is afraid or intimidated by the perpetrator, despite being aware of the abusive situation [103, 104]. A study carried out in Ireland reported in particular that societies could recognize a context of violence as such, whereas the victim, especially after many years of abuse and intimidation, could accept violent behaviour perpetrated within a familial relationship [105]. In other words, it is very difficult to distinguish a legitimate/consensual financial transaction from an abusive/exploitative one. Older persons indeed might also want to compensate financially relatives who take care of them [106]http://www.ncbi.nlm.nih.gov/books/NBK98784/-ch13.r50. Moreover, the cultural context in which financial abuse takes place should be considered, and thus the possibility that elderly persons financially support family members facing economic difficulties could be approved in terms of intergenerational solidarity and ‘societal expectation’. It should be noted however, that this attitude is not recognized in all national cultures [102, 107].

#### Health

Our results showed that with the increase of somatic and anxiety symptoms, the probability of being abused grew too. These associations suggest that, in general, the poor health conditions of older people may imply an increased dependency on others, placing them at risk of abuse. Previous observations did indeed indicate that disability and dependency due to physical and cognitive problems increase the risk of the elderly becoming victims of mistreatment [44, 79, 108]. Literature also showed the positive link between depression, anxiety and somatic symptoms, with specific regard to older persons [109, 110]. Various studies have revealed that symptoms such as anxiety and unhappiness experienced by the victims of elder abuse are more likely to be consequences of maltreatment rather than potential risk factors [111, 112]. Further studies highlighted, mainly concerning women, a positive association between abuse (e.g. IPV and psychological) and somatic complaints [113, 114], although further aspects such as depression and anxiety play a role in this respect [115].

With specific regard to men, it has been observed that their mental health problems are often under-diagnosed and under-treated in most European countries [116]. Men are indeed less likely than women to report cognitive symptoms and somatic complaints, and to seek treatment for such complaints. Men seem to feel the pressure to be strong, to be ‘man-like’ with ‘male-appropriate attitudes and behaviour’, and this could represent a dangerous substrate for elder abuse especially in later life, when men are more frail and vulnerable [117]. In addition, older men suffering from physical/mental health problems, including anxiety, are at risk of self-neglect, and this could in turn result in a higher risk of victimization [43].

### Community Level

#### Profession

Low white-collar workers were less abused than blue-collar workers. These results are similar to those from a previous study concerning men’s experiences of lifetime violence (mainly aggressive language and physical assaults) in Stockholm [20], showing that men aged 18–64 years, particularly blue-collar workers, were often victims of violence occurring mostly in public settings and workplace. Workplace violence thus seems common, but it varies in frequency depending on the type of job [118]. We may speculate that blue-collar workers are employed in more abusive and less protected/regulated workplaces, where they are at high risk of poor work supervision or lack it altogether [20]. Previous research also indicates that individuals with low labour positions often work in areas where violence is more usual and frequent [119–121].

#### Social support

Increase in social support was related to a reduced probability of being abused. A low level of social support and social isolation may indeed represent crucial risk factors for elder abuse. According to some authors, associations between different types of elder abuse and low levels of social support have been found [45, 122]. Conversely, a high level of social support seems to be a protective factor in reducing the risk of elder mistreatment [74], also decreasing depression in old age as a possible additional risk factor for elder abuse [26]. Other studies have shown the potential of affective solidarity from family as support for psychological symptoms [123, 124].

With specific regard to gender, men seem less likely to receive social support from informal networks than women. Women show a lifestyle more linked to social relations, and appear to benefit from support from multiple sources, whereas men rely mainly on spouses/partners [125]. For many older men, the spouse/partner is the principal or unique confidant, and when a man becomes a widower he loses his most important relationship, whereas older women rely on different close relationships such as other relatives [126]. Overall, men show a lower level of social engagement, due to their prevalent reliance on their spouses/partners for social support, and consequently lower participation in social activities [127]. Older men also experience lower levels and frequency of involvement in social life due to health (physical and cognitive) limitations [128]. On the whole, a greater perceived social support for women is reported [129]. When social support is available, men seem to derive greater health benefits from social networks than women [27–28], and this in turn decreases the probability of their being abused.

It is important to keep in mind that apparent self-neglect situations regarding older men may not be the consequence of a personal choice of not caring for themselves, as is usually perceived to be the male stereotype. Such a context may, in fact, indicate a lack of support from the family, also in terms of abandonment and neglect [15]. Stratton and Moore [130] highlighted the effects of past ‘fractured’ relationships between older men and their family members as potential driving forces towards lack of support and related episodes of mistreatment, with the consequence of an increased risk of neglect of elderly men [11]. Older men and their adult children seem therefore to face many difficulties in promoting family cohesion, the sense of family ties and obligations, and in repairing family breakdown when the older person needs support [131].

### Future Research Directions

Although experiences of men’s violence are emerging in the literature, and our study on elder abuse has provided some new insights into this issue, more extensive and specific research is necessary. It is essential that future studies focus on elder abuse in both genders, in order to raise awareness of existing mistreatment of older men. There are indeed still strong social/cultural norms which prevent men acknowledging victimization [38–40], although ageing and ageism can expose in general older persons to episodes of violence and neglect, especially when the persons in later life are disabled and depend on others for assistance in carrying out daily activities.

Future research should also explore in more depth men and women as both victims and perpetrators, in addition to exploring the perception of support from family and social relationships. In particular, more research focusing on the impact of mistreatment on men’s health is crucially needed, as is the exploration of pathways by which community-societal level factors are linked with individual ones and their impact on male elder abuse. Finally, studies are needed to evaluate in more depth the multilevel approach, in an Ecological Model framework for abuse of older men. This model has been applied to female victims of abuse, as previously mentioned [64, 132] and it shows potential to give a valid theoretical picture also of male abuse prevention.

### Limitations

This study has some limitations [45]. First, data are derived only from large urban centres in seven European countries and based on self-reports by older participants, and as a result are subject to possible recall bias. Moreover, the study excluded elderly persons with cognitive impairment (who were not able to appropriately complete the survey). Both of these aspects impact the degree to which the findings can be generalized. Second, the relatively low numbers of participants who reported some types of abuse episodes (e.g. injury and sexual abuse), which could be linked to systematic under-reporting of abuses, warrants caution in the interpretation of findings, and this has further impacts upon ability to generalize. Third, the data are cross-sectional, which does not permit the establishment of causal links between variables. Future research in this area will require a longitudinal design in order to test the relation found between various dimensions and elder abuse.

Despite these limitations, our study provides the following benefits: cross-national data on various aspects of elder abuse, in particular against older men, where this specific aspect still represents an under-investigated issue; a workable definition of abuse (including injuries) and validated instruments to assess this phenomenon; findings and tools which could be used by policy makers, clinicians and researchers at both Europe-wide and national levels for a range of activities (e.g. monitoring abuse, awareness campaigns).

## Conclusions

This study evidences the fact that male elder abuse exists but is still is under-recognized. The study also confirms what previous literature has already highlighted [10, 20, 43, 133], namely that the usual socially-constructed meaning of gender, which considers males as violent and women as pacific, is rather anachronistic. Thus, we can, with confidence, indicate that men may be as exposed to abuse as women are. These results break down the erroneous belief that elder abuse is a female question.

This study further found that exposure to abuse among older men is associated with various factors. Some factors pertain also to women (e.g. fragile older age conditions), and others are more gender-specific in different cultures and countries, in particular attitudes, beliefs and behaviours related to the insecurity/vulnerability of older men as dependent on spouse and/or children for several needs (e.g. housing). Moreover, social/cultural norms supporting traditional male stoicism and self-reliance may prevent older men from reporting abuse and seeking help.

This paper in particular focused on the contextual risk factors using an applied Ecological Model, which is a useful framework for understanding male elder abuse and for providing recommendations for the development of community-based prevention/educational programs and inter-professional/collaborative interventions [58, 134]. The Ecological Model allows for an integrated and holistic approach to the prevention of violence, through the framework of nested systems linking elder abuse to broader social issues, and taking into account different levels of interactions (individual, relationship, community and society) [135]. Specifically, individual prevention strategies can promote attitudes, beliefs, and behaviours that may prevent elder abuse [58], but it is also crucial to analyse the abuse of older men as a societal problem, as well as an individual and family question [71].
